# Effect of Exercise-Induced Reductions in Blood Volume on Cardiac Output and Oxygen Transport Capacity

**DOI:** 10.3389/fphys.2021.679232

**Published:** 2021-05-31

**Authors:** Janis Schierbauer, Torben Hoffmeister, Gunnar Treff, Nadine B. Wachsmuth, Walter F. J. Schmidt

**Affiliations:** ^1^Department of Sports Medicine/Sports Physiology, University of Bayreuth, Bayreuth, Germany; ^2^Department of Exercise Physiology and Metabolism, University of Bayreuth, Bayreuth, Germany; ^3^Institute of Applied Training Science, Leipzig, Germany; ^4^Division of Sports and Rehabilitation Medicine, University of Ulm, Ulm, Germany

**Keywords:** stroke volume, heart volume, hemoglobin concentration, peripheral oxygen saturation, arterial oxygen content

## Abstract

We wanted to demonstrate the relationship between blood volume, cardiac size, cardiac output and maximum oxygen uptake ($\dot{V}$O_2max_) and to quantify blood volume shifts during exercise and their impact on oxygen transport. Twenty-four healthy, non-smoking, heterogeneously trained male participants (27 ± 4.6 years) performed incremental cycle ergometer tests to determine $\dot{V}$O_2max_ and changes in blood volume and cardiac output. Cardiac output was determined by an inert gas rebreathing procedure. Heart dimensions were determined by 3D echocardiography. Blood volume and hemoglobin mass were determined by using the optimized CO-rebreathing method. The $\dot{V}$O_2max_ ranged between 47.5 and 74.1 mL⋅kg^–1^⋅min^–1^. Heart volume ranged between 7.7 and 17.9 mL⋅kg^–1^ and maximum cardiac output ranged between 252 and 434 mL⋅kg^–1^⋅min^–1^. The mean blood volume decreased by 8% (567 ± 187 mL, *p* = 0.001) until maximum exercise, leading to an increase in [Hb] by 1.3 ± 0.4 g⋅dL^–1^ while peripheral oxygen saturation decreased by 6.1 ± 2.4%. There were close correlations between resting blood volume and heart volume (*r* = 0.73, *p* = 0.002), maximum blood volume and maximum cardiac output (*r* = 0.68, *p* = 0.001), and maximum cardiac output and $\dot{V}$O_2max_ (*r* = 0.76, *p* < 0.001). An increase in maximum blood volume by 1,000 mL was associated with an increase in maximum stroke volume by 25 mL and in maximum cardiac output by 3.5 L⋅min^–1^. In conclusion, blood volume markedly decreased until maximal exhaustion, potentially affecting the stroke volume response during exercise. Simultaneously, hemoconcentrations maintained the arterial oxygen content and compensated for the potential loss in maximum cardiac output. Therefore, a large blood volume at rest is an important factor for achieving a high cardiac output during exercise and blood volume shifts compensate for the decrease in peripheral oxygen saturation, thereby maintaining a high arteriovenous oxygen difference.

## Introduction

Over the years, it has been well established that cardiac output ($\dot{Q}$) is a major limiting factor for the maximum oxygen uptake ($\dot{V}$O_2max_) during exercise (Bassett and Howley, 2000; Stöhr et al., 2011; Lundby et al., 2017). $\dot{Q}$ equals the product of heart rate (HR) and stroke volume (SV), and is largely determined by a harmonic structural and functional adaption of the heart, where especially cardiac compliance is a prerequisite for large end-diastolic volumes (Levine, 2008; Magder, 2016). If these prerequisites are given, the heart, e.g., the athletic heart—which is characterized by greater dimensions, specifically harmonically increased left-ventricular dimensions (Baggish and Wood, 2011; La Sanz-de Garza et al., 2020)—has a greater ability to use the Frank-Starling-mechanism. This well described mechanism allows for an efficient realization of SV due to a preload mediated stretch of the myocardium (Woodiwiss and Norton, 1995). The preload largely depends on the circulating blood volume (BV), which therefore plays a key role for cardiac function. Unsurprisingly, previous studies revealed a strong positive relationship between BV and $\dot{V}$O_2max_, which is mainly attributable to an increased venous filling resulting in a larger $\dot{Q}$_max_ (Hellsten and Nyberg, 2015). This effect has been demonstrated in cross-sectional (González-Alonso et al., 2000; Warburton et al., 2002) and longitudinal, i.e., manipulative studies (Wilmore et al., 2001; Daussin et al., 2007; Bonne et al., 2014).

Noteworthy, BV has been shown to decrease during incremental exercise to exhaustion, due to a reduction in plasma volume by approx. 10%, however, SV was not measured (Kawabata et al., 2004),. Consequently, this exercise mediated BV reduction might impair venous return during moderate and intensive exercise and exert a limiting effect on SV and, according to the Fick principle, $\dot{V}$O_2max_. On the other hand, the concomitant increase in hemoglobin concentration [Hb] factually augments O_2_ transport capacity. This would allow to mitigate the exercise induced arterial O_2_ desaturation (Gaston et al., 2016; Goodrich et al., 2018) and thereby enabling a sufficient arterio-venous oxygen difference during exercise at high intensities. Moreover, there seems to be an interaction between $\dot{Q}$_max_ and the arterial oxygen content (CaO_2_), as recent studies have demonstrated a regulatory effect of the CaO_2_ on the $\dot{Q}$ response during dynamic exercise (Roach et al., 1999; Calbet, 2000).

Interestingly, percentage changes in BV during exercise have been evaluated before (Dill and Costill, 1974), but data on absolute changes are scarce. Therefore, the effects of temporary BV changes during exercise on oxygen transport and endurance performance have not yet been fully elucidated. The first aim of this study was to quantify absolute BV changes during moderate and maximal exercise and to calculate their possible influence on SV, $\dot{Q}$, and $\dot{V}$O_2_, while evaluating structural cardiac variables. Second, we wanted to quantify the scale of changes in CaO_2_ due to the combined effects of hemoconcentration and exercise-induced oxygen desaturation. Using these data, we aimed to calculate whether the potentially beneficial effects of hemoconcentration for oxygen transport might compensate or even exceed the detrimental effects of a diminished BV on the SV and the decreasing arterial oxygen desaturation during exercise.

## Materials and Methods

### Participants

Twenty-four healthy, non-smoking and very heterogeneously trained males were included in the study (see Table 1 for subject characteristics). We explicitly focused on selecting participants with a wide range of performance status to compare the expected different structural and functional properties. The participants provided written consent after they were informed about the content of the study, the associated risks and the possibility to withdraw without indication of any reason. The study was approved by the ethics committee of the University of Bayreuth in Germany.

**TABLE 1:** Subject characteristics.

*n* = 24	**Mean ± SD**	**Min.**	**Max.**
Age (y)	27.0 ± 4.6	20	43
Height (cm)	183.5 ± 6.5	171	199
Body mass (kg)	77.8 ± 8.5	67.4	106.6
BMI (kg⋅m^–2^)	23.1 ± 1.7	19.6	26.9
Lean body mass (kg)	68.2 ± 6.8	56.0	89.1
Fat mass (%)	12.3 ± 3.8	5.2	21.9
Ferritin (μg⋅L^–1^)	129 ± 53	41	240

**The data are presented as the mean values ± standard deviations. Min, minimum; Max, maximum; BMI, body mass index*.*

### Study Design

Participants performed two cycle ergometer tests on consecutive days. During the first ergometer test, the maximum oxygen uptake ($\dot{V}$O_2max_) was determined. The second test followed a similar protocol and was performed to measure SV and $\dot{Q}$ at 60% and near 100% of the first test’s maximum power (P_max_) using an inert gas rebreathing method. Simultaneously, hemoglobin concentration for the calculation of BV (see Equation 2) and peripheral hemoglobin-O_2_ saturation (SpO_2_) were determined. Prior to the performance tests, anthropometric measurements including body composition were conducted using bioelectrical impedance analysis. A cubital venous blood sample was drawn to determine basic hematological parameter and ferritin levels in order to exclude any iron deficiencies. The heart dimensions were determined by 3D echocardiography. BV and Hbmass were measured twice on consecutive days using the optimized CO-rebreathing method (Schmidt and Prommer, 2005; Prommer and Schmidt, 2007). All measurements took place within 7 days and, except for the echocardiography, were carried out under standardized environmental conditions in our laboratory (room temperature: 21.5°C, barometric pressure: ∼736 mmHg).

### Analytical Procedures and Echocardiography

Lean body mass and fat mass were measured twice consecutively using bioelectrical impedance analysis (InBody 720, InBody Co., Seoul, South Korea). Cubital venous blood samples (8 mL) were taken after the participants rested for 15 min in a seated position. Heparinized blood samples were analyzed using a fully automated hematology system (Sysmex XN 1000-1-A, Sysmex, Norderstedt, Germany) for basic hematological parameters. In the serum, the ferritin concentration and C-reactive protein (CRP) level were determined by enzyme immunoassays [ferritin: LKFE1, ELISA, and Immulite 1000 (Siemens Healthcare Diagnostics GmbH, Germany); CRP: highly sensitive—LKCRP1, ELISA, and Immulite 1000 (Siemens Healthcare Diagnostics GmbH, Germany)]. Transthoracic echocardiography was conducted using a commercially available cardiology ultrasound system (Philips EPIC 7, Phillips Medical Systems, Andover, MA, United States) with a 1.0–5.0 MHz sector array transducer (Philips S5-1, Phillips Medical Systems, Andover, MA, United States) to measure both the end-systolic and end-diastolic volumes, left ventricular muscle mass (LVMM), left ventricular end-diastolic diameter (LVEDD) and SV at rest. Heart volume (HV) was determined according to the methods described by Dickhuth et al. (1990).

### Incremental Ergometer Tests

Aerobic performance was determined using an incremental protocol on a cycle ergometer (Excalibur, Lode, Groningen, Netherlands). After a 3-min warm-up phase of 100 Watts, the mechanical power output was continuously increased by 50 Watts every 3 min (stepwise by 17, 17, and 16 Watts per minute) until subjective exhaustion was reached. $\dot{V}$O_2_ was determined via breath-by-breath technology (Innocor system, Innovision, Glamsbjerg, Denmark) and calculated as the mean value across the last 30 s before exhaustion. Capillary blood samples were taken from a hyperemized earlobe to quantify the lactic acid concentrations before exercise, every 3 min during exercise, immediately at exhaustion and 1, 3, 5, and 7 min after exhaustion (Biosen S-Line, EKF-Diagnostic, Barleben, Germany). During the second test, capillary blood samples were taken for the measurement of [Hb] (HemoCue 201, Hemocue AB, Ängelholm, Sweden) at 60% and near 100% of the first test’s P_max_ to calculate changes in BV during the exercise period. Peripheral oxygen saturation (SpO_2_) was continuously determined by finger pulse oximetry (9590 oximeter, Nonin Medical Inc., Plymouth, United States) and arterial oxygen content (CaO_2_) was calculated according to formula 1:

(1) $$CaO_{2}\left(mL\cdot dL^{-1}\right)=\left[Hb\right]\left(g\cdot dL^{-1}\right)\times SpO_{2}\left(\%\right)÷100\times 1.39$$

### Measurement of Cardiac Output, Stroke Volume, and Arteriovenous Oxygen Difference During Exercise

SV, $\dot{Q}$, and avDO_2_ were determined by inhaling a mixture of oxygen and two inert gases, i.e., nitrogen oxide (N_2_O, 0.5%) and sulfur hexafluoride (SF_6_, 0.1%) with a photoacoustic gas analyzer (Innocor system, Innovision, Glamsbjerg, Denmark). Because this rebreathing maneuver might impair test performance, two tests were performed: one for the determination of $\dot{V}$O_2max_ and one for the determination of $\dot{Q}$. The starting points for the rebreathing measurements during the second test were at 60% P_max_ and the last fully completed 1-min increment from the first ergometer test, respectively. Both rebreathing procedures took place after 10 s in the respective stages. Each rebreathing process lasted approximately 10 s and consisted of five respiratory cycles. $\dot{Q}$ was then derived from the changes in the concentrations of the inert gases in the expiratory volumes. SV was calculated on the basis of $\dot{Q}$ and heart rate (HR), and avDO_2_ was calculated by applying the Fick equation. The typical error of this method in our lab was 4.9% at 200 Watts, which is in line with earlier research (Fontana et al., 2009).

### Determination of Hemoglobin Mass and Total Blood Volume

At least 2 h after the incremental test, when the plasma volumes had returned to pre-exercise values (Schmidt et al., 1990), Hbmass, total blood (BV), plasma (PV) and erythrocyte (RCV) volumes were determined using the optimized CO-rebreathing method according to the methods reported by Schmidt and Prommer (2005), Gore et al. (2006), and Prommer and Schmidt (2007). In brief, an individual dose of carbon monoxide (CO; 0.8–1.0 mL⋅kg^–1^) was administered and rebreathed along with 3 L of pure oxygen for 2 min. Capillary blood samples were taken before and 6 and 8 min post-administration of the CO dose. The blood samples were analyzed for the determination of %HbCO using an OSM III hemoximeter (Radiometer, Copenhagen, Denmark). Hbmass was calculated on the basis of the mean change in %HbCO before and after the CO was rebreathed. BV was then calculated according to formula 2:

(2) $$B\,V\,\left(m\,L\right)=H\,b\,m\,a\,s\,s\,\left(g\right)\times 100÷{\left[H\,b\right]}_{\text{ven}}\,\left(g\,\text{⋅}\,d\,L^{-1}\right)\times F^{-1}$$

where [Hb]_ven_ = venous hemoglobin concentration and F = cell factor at sea level (Fricke, 1965). For the calculations of BV during exercise, capillary [Hb] was measured during the second cycle ergometer test and modified for the venous conditions (Patel et al., 2013). Since the Hbmass does not change over short periods of time (Eastwood et al., 2008), the temporally offset determination of the [Hb] for the calculation of the BV is possible without compromising accuracy. For a detailed description and the accuracy of the methods see Schmidt and Prommer (2005), Gore et al. (2006), and Prommer and Schmidt (2007). In our laboratory, the typical error for Hbmass is 1.5%, which is comparable to previous investigations (Hütler et al., 2000; Robertson et al., 2010), while the typical error for BV is 2.5%.

### Statistical Analysis

The data are presented as the arithmetic means and standard deviations. Statistical analysis was conducted using IBM SPSS Statistics 26 (IBM, Armonk, United States). A one-way ANOVA with repeated measures followed by *post hoc* tests with Bonferroni correction were performed to find significant differences between the resting, submaximal exercise and maximal exercise conditions. Simple linear regression and correlation analysis was performed to assess the relationships between two variables. The level of significance was set to *p* ≤ 0.05 (^∗^).

## Results

As a result of the intended heterogeneity of the participants, absolute and relative performance as well as hematological and cardiac characteristics showed large interindividual variability (Table 2). Relative values for $\dot{V}$O_2max_ ranged between 47.5 and 74.1 mL⋅kg^–1^⋅min^–1^. BV and Hbmass ranged between 75 and 101 mL⋅kg^–1^ and 10.6 and 14.2 g⋅kg^–1^, respectively. HV ranged between 7.7 and 17.9 mL⋅kg^–1^. Left ventricular end diastolic diameter (LVEDD) and muscle mass (LVMM) ranged between 45 and 63 mm and 116 and 303 g, respectively.

**TABLE 2:** Performance and cardiological data and hemoglobin mass.

	**Mean ± SD**	**Min.**	**Max.**
P_max_ (W)	377 ± 48	267	450
$\dot{V}$O_2max_ (mL⋅min^–1^)	4,624 ± 484	3,481	5,520
$\dot{V}$O_2max_ (mL⋅min^–1^⋅kg^–1^)	59.8 ± 6.6	47.5	74.1
RER_max_	1.2 ± 0.1	1.1	1.4
[Lac]_max_ (mmol⋅L^–1^)	13.5 ± 2.4	9.2	17.2
Hbmass (g)	980 ± 124	746	1,365
Hbmass (g⋅kg^–1^)	12.6 ± .1.1	10.6	14.2
ESV (mL)	77.9 ± 21.4	31	119
EDV (mL)	172.9 ± 34.1	86	249
HV (mL)	1,101 ± 213	560	1,575
HV (mL⋅kg^–1^)	14.2 ± 2.2	7.7	17.9
LVEDD (mm)	52.4 ± 4.6	45	63
LVMM (g)	184 ± 49	115	303

**The data are presented as the means ± standard deviations. Min., minimum; Max., maximum; P, power; $\dot{V}$O_2m*ax*_, maximum oxygen uptake; RER_*max*_, maximum respiratory exchange ratio; [Lac]_*max*_, maximum lactate concentration; Hbmass, hemoglobin mass; ESV, endsystolic volume; EDV, enddiastolic volume; HV, heart volume; LVEDD, left ventricular enddiastolic diameter; LVMM, left ventricular muscle mass*.*

### Exercise Study

**FIGURE 1:**
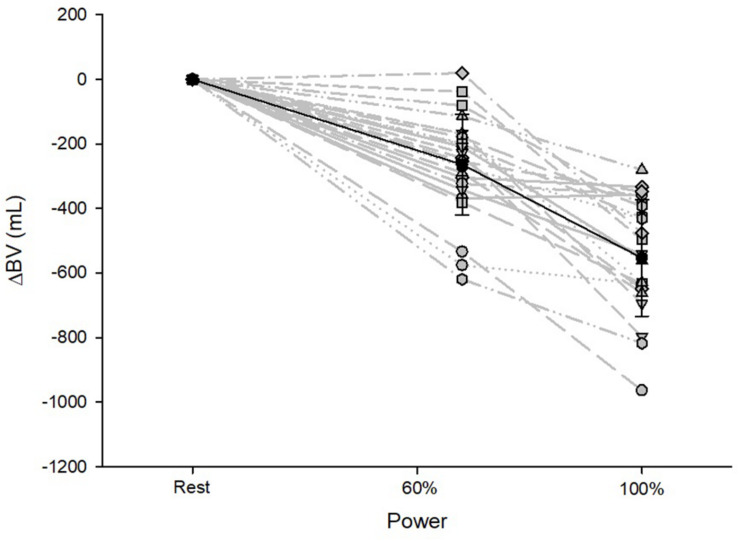
Changes in BV from resting to submaximal (60% P_max_) and maximal exercise (100% P_max_) conditions. Data are presented as the mean with standard deviations.

BV decreased until maximum exercise by 8% (567 ± 187 mL, *p* < 0.001, Figure 1). There was a moderate correlation between the BV at rest (BV_rest_) and the amount of intravasal fluid lost until maximum exercise (*r* = 0.47, *p* = 0.04); however, when percentage changes were calculated, no such relationship exists. Mean SV significantly increased from resting to submaximal exercise by 54 ± 20 mL (*p* < 0.001) with no significant difference in the further course until maximum exercise. However, large variability in the SV response was detected, especially from submaximal to maximal exercise with athletes demonstrating a progressive increase, plateau formation or a drop in SV. There was no correlation between the SV response from submaximal to maximal conditions and relative VO_2max_, BV_max_ and the amount of BV shifted in the same time interval. HR continuously increased until maximal exercise, as did submaximal and maximal avDO_2_ and $\dot{Q}$ (Table 3). [Hb] significantly increased until maximum exercise by 8.5% (1.3 ± 0.4 g⋅dL^–1^, *p* < 0.001), while the SpO_2_ level in the capillary blood decreased by approximately 6.1% (*p* = 0.001, Table 3). Arterial oxygen content (CaO_2_) remained unchanged between resting, submaximal and maximal exercise conditions (Table 3).

**TABLE 3:** Cardio-circulatory data at rest, 60% of P_max_ and P_max_ (*n* = 24).

	**$\dot{V}$O_2_ (mL**⋅**min**^–^**^1^)**	**$\dot{Q}$ (L**⋅**min**^–^**^1^)**	**SV (mL)**	**HR (bpm)**	**avDO_2_ (mL**⋅**dL**^–^**^1^)**	**[Hb]_ven_ (g**⋅**dL**^–^**^1^)**	**SpO_2_ (%)**	**CaO_2_ (mL**⋅**dL**^–^**^1^)**	**$\dot{Q}$ x CaO_2_ (mL⋅min**^–^**^1^)**
Rest	–	6.2 ± 1.8	94 ± 15.2	65 ± 11.1	–	15.0 ± 0.7	97.5 ± 0.7	20.3 ± 0.7	1,259 ± 264
60% P_max_	3,150 ± 350	21.0 ± 3.1***	150 ± 27.4***	142 ± 11.0***	15.0 ± 1.2	15.5 ± 0.5***	94.5 ± 2.1***	20.4 ± 0.8	4,284 ± 621***
P_max_	4,624 ± 484^#^	26.1 ± 4.4***^/#^	147 ± 30.4***	189 ± 8.7***^/#^	17.9 ± 1.7^#^	16.2 ± 0.6***^/#^	91.4 ± 3.2***^/#^	20.6 ± 1.1	5,376 ± 790***^/#^

The data are presented as the means ± standard deviations. P, power; $\dot{V}$O_2_, oxygen uptake; $\dot{Q}$, cardiac output; SV, stroke volume; HR, heart rate; avDO_2_, arteriovenous oxygen difference; CaO_2_, arterial oxygen content; SpO_2_, peripheral oxygen saturation; [Hb]_ven_, venous hemoglobin concentration. Significant differences compared to the resting values are indicated as follows: ***significant compared to resting values (*p* < 0.001), ^#^significant compared to 60% P_max_ (p < 0.05).

### Regression Analysis

#### Maximum Oxygen Uptake

**FIGURE 2:**
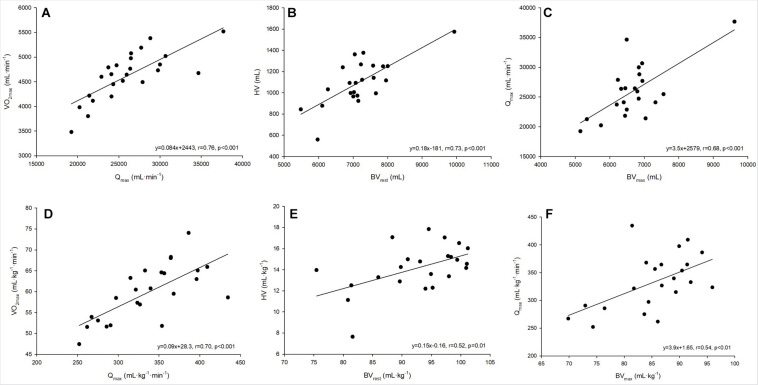
**(A–F)** Linear regressions analysis between absolute and relative values for maximum oxygen uptake and maximum cardiac output **(A,D)**, heart volume and blood volume at rest **(B,E)** and maximum cardiac output and blood volume at maximum exercise **(C,F)**.

A strong linear correlation was found between absolute (*p* < 0.001) and relative (*p* < 0.001) values for $\dot{V}$O_2max_ and $\dot{Q}$_max_ (Figure 2A). The slope of the respective regression line indicates that an increase in $\dot{Q}$_max_ by 1 L between different individuals was associated with an increase in $\dot{V}$O_2max_ by 84 mL⋅min^–1^. $\dot{V}$O_2max_ was also strongly correlated to BV_rest_ and BV_max_, while the correlation to BV_max_ tended to be slightly stronger than that to BV_rest_ (*r* = 0.69 vs. *r* = 0.65, both *p* < 0.001, Table 4). The correlation between $\dot{V}$O_2max_ and avDO_2_ was not significant, while the anatomical parameters HV, LVMM and Hbmass also significantly correlated with $\dot{V}$O_2max_ (all *p* < 0.05, see Table 4). When the abovementioned variables were normalized to both body mass and lean body mass, the correlations were still significant (see Table 4).

**TABLE 4:** Pearson’s product-moment correlations (*r*) and level of significance (*p*) between the absolute and (lean) body mass normalized $\dot{V}$O_2max_ and cardio-circulatory variables.

		**Absolute**		**Relative**			
			
				**kg**^–^**^1^**		**kg**^–^**^1^ LBM**	
					
		***r***	***p***	***r***	***p***	***r***	***p***
$\dot{V}$O_2max_ (L⋅min^–1^)	$\dot{Q}$_max_ (L⋅min^–1^)	0.76	<0.001	0.75	<0.001	0.66	<0.001
	SV_max_ (mL)	0.71	<0.001	0.72	<0.001	0.55	0.005
	HV (mL)	0.68	0.002	0.46	0.04	0.45	0.03
	LVEDD (mm)	0.30	0.18	0.56	0.006	0.47	0.03
	LVMM (g)	0.58	0.003	0.43	0.03	0.38	0.06
	Hbmass (g)	0.67	0.002	0.59	0.008	0.43	0.04
	BV_rest_ (mL)	0.65	0.001	0.63	0.001	0.40	0.06
	BV_max_ (mL)	0.69	0.002	0.61	0.004	0.45	0.04
	avDO_2max_ (mL⋅dL^–1^)	−0.08	0.71	0.03	0.90	0.04	0.87
	ΔSpO_2max_ (%)	−0.42	0.04	−0.37	0.08	−0.29	0.17
	HR_max_ (bpm)	−0.14	0.51	−0.35	0.09	−0.38	0.07
$\dot{Q}$_max_ (L⋅min^–1^)	HV (mL)	0.64	0.001	0.49	0.02	0.49	0.02
	BV_rest_ (mL)	0.64	0.001	0.50	0.01	0.42	0.04
	BV_max_ (mL)	0.68	0.001	0.54	0.009	0.46	0.03
	SV_max_ (mL)	0.96	<0.001	0.94	<0.001	0.93	<0.001
	ΔSpO_2max_ (%)	−0.58	0.005	−0.66	<0.001	−0.57	0.005
	HR_max_ (bpm)	−0.53	0.007	–	–	–	–
	LVMM (g)	0.44	0.03	0.25	0.26	0.21	0.33
SV_max_ (mL)	BV_rest_ (mL)	0.65	0.001	0.45	0.03	0.40	0.05
	BV_max_ (mL)	0.70	<0.001	0.53	0.01	0.49	0.02
	HV (mL)	0.66	0.001	0.50	0.01	0.51	0.01
	LVEDD (mm)	0.43	0.004	0.31	0.15	0.25	0.24
	LVMM (g)	0.52	0.01	0.32	0.14	0.32	0.13
HV (mL)	BV_rest_ (mL)	0.73	0.002	0.52	0.02	0.55	0.007
	BV_max_ (mL)	0.77	<0.001	0.61	0.03	0.65	0.001
	LVEDD (mm)	0.61	0.003	0.36	0.10	0.37	0.08
LVEDD (mm)	BV_rest_ (mL)	0.63	0.001	0.55	0.006	0.51	0.01
	BV_max_ (mL)	0.58	0.006	0.50	0.02	0.25	0.05

**LBM, lean body mass; $\dot{V}$O_2max_, maximum oxygen uptake; $\dot{Q}$_rest_, cardiac output at rest; $\dot{Q}$_max_, maximum cardiac output; SV_rest_, stroke volume at rest; SV_max_, maximum stroke volume; HV, heart volume; Hbmass, hemoglobin mass; BV_max_, blood volume at maximum exercise; avDO_2max_, arteriovenous oxygen difference at maximum exercise; LVEDD, left ventricular end-diastolic diameter; LVMM, left ventricular muscle mass; SpO_2max_, peripheral oxygen saturation at maximum exercise*.*

#### Cardiac Output and Stroke Volume in Relation to Blood Volume and Cardiac Size

The cross-sectional data show that $\dot{Q}$_max_ was strongly correlated with SV_max_ but was also negatively correlated with HR_max_ (see Table 4). SV_max_ was similarly correlated to HV and BV_max_ (Table 4). According to the slope of the regression line, an increase in BV_max_ by 1,000 mL was associated with an increase in SV_max_ by 25.1 mL, and an increase in HV by 100 mL increased SV_max_ by 6 mL. BV_rest_ was significantly correlated with HV (*p* = 0.002, Figure 2B), indicating that an increase in BV_rest_ by 1,000 mL was associated with an increase in HV by 179 mL. Concerning the significant correlation between BV_max_ and $\dot{Q}$_max_ (*p* < 0.001, Figure 2C), an increase in BV_max_ by 1,000 mL was associated with an increase in $\dot{Q}$_max_ by 3.5 L⋅min^–1^.

Applying these cross-sectional data to the intra-individual changes in BV during exercise, the 8% reduction found in this study would lead to a decrease in $\dot{Q}$_max_ by 1.6 L⋅min^–1^ and thus 133 mL⋅min^–1^ in $\dot{V}$O_2max_.

LVEDD and LVMM were significantly correlated with BV_max_, SV_max_ and Q_max_. SpO_2_ was not correlated to the absolute values of $\dot{Q}$_max_; however, there were, however, significant correlations to $\dot{Q}$_max_ when normalized to body mass and lean body mass (see Table 4).

## Discussion

The main goals of this study were (i) to determine the correlations between BV, HV, $\dot{Q}$_max_ and $\dot{V}$O_2max_, (ii) to quantify the BV shifts occurring during submaximal and maximal exercise and (iii) to evaluate the effects of these volume shifts on $\dot{Q}$_max_ and arterial oxygen transport capacity in healthy heterogeneously trained males. The major findings of the cross-sectional study showed that an elevated BV_max_ by 1,000 mL was associated with an increase in SV_max_ by approximately 25 mL, leading to an increase in $\dot{Q}$_max_ by approximately 3.5 L⋅min^–1^. As BV significantly decreased from rest to maximal exercise by 567 mL (8%), we calculate a possible limitation in $\dot{Q}$_max_ by 1.6 L⋅min^–1^, but we also demonstrate a consistent oxygen transport capacity due to the increase in hemoconcentration.

### Stroke Volume and Heart Rate

SV is the most important, strongly blood volume-dependent component of the cardio-circulatory response during exercise (Munch et al., 2014). In our study, SV increased by 60% from resting to submaximal exercise conditions. These values are higher than those that were reported during progressive exercise in healthy, untrained subjects (Higginbotham et al., 1986; Rowland, 2005, 2009) and lower than those reported in highly trained endurance athletes (Ekblom and Hermansen, 1968). According to Vella and Robergs (2005) there are four main types of SV responses with increasing exercise intensity. While the mean values for SV in this study showed no significant changes from submaximal to maximal exercise, substantial variability was found among subjects, indicating a progressive increase, plateau formation or a decrease in SV on the individual level. These variations in the SV response from 60 to 100% P_max_ were not correlated to VO_2max_.

HR_max_ was negatively related to $\dot{V}$O_2max_ and $\dot{Q}$_max_ (Table 4), which corresponds with previous results (Rowell, 1986). With regard to the strong correlation between $\dot{Q}$_max_ and SV_max_, these findings demonstrate that, at least near maximal performance, inter-individual differences in $\dot{Q}$ result primarily from variations in SV in both trained and untrained subjects.

### Blood Volume and Cardiac Output

As demonstrated by Bonne et al., early increases in $\dot{V}$O_2max_ following 6 weeks of endurance training are reverted to pretraining levels when the training-induced gains in BV are eliminated by means of phlebotomy, which indicates that total BV is the most important factor of the SV response in early adaptations (Bonne et al., 2014). However, in a recent study it was demonstrated that the increase in $\dot{V}$O_2peak_ following 10 weeks of endurance training was preserved even after Hbmass and BV were reversed to pretraining levels by phlebotomy, thereby contradicting the assumption that improvements in $\dot{V}$O_2peak_ and $\dot{Q}$_peak_ are exclusively attributed to BV expansion (Skattebo et al., 2020). In their comprehensive review, Lundby et al. (2017) revealed the underlying mechanisms of improvements in $\dot{V}$O_2max_. Increases in SV at rest and during exercise are mainly dependent on increased venous return as a result of a high BV leading to higher end-diastolic volumes via the Frank-Starling mechanism. As we found significant correlations between $\dot{Q}$_max_ and BV_max_ (Figures 2C,F), we hereby confirmed these mechanisms for our heterogeneously trained population included in this study. Moreover, we demonstrated the quantitative effect of an expanded BV: Male subjects with a BV of ∼5 L are able to achieve a $\dot{Q}$_max_ of ∼20 L⋅min^–1^. A change in BV by 1 L was related to a change in $\dot{Q}$_max_ by ∼3.5 L⋅min^–1^. This indicates that when the BV is increased, e.g., by 2.5 L as a result of a higher training status (Heinicke et al., 2001), $\dot{Q}$_max_ is increased considerably to 28.8 L⋅min^–1^. As an increase in $\dot{Q}$_max_ by 1 L is associated with an increase in $\dot{V}$O_2max_ by ∼84 mL⋅min^–1^, information on increases in BV is particularly important. All these considerations are also valid for the relative values and prove a mechanism that is independent of the anthropometric conditions (see Figures 2D–F and Table 4). From a practical point of view, the question remains whether changes in BV caused by environmental conditions influence $\dot{Q}$_max_ and therefore $\dot{V}$O_2max_. At high altitude, a lower $\dot{Q}$_max_ also refers to a plasma volume reduction (Siebenmann et al., 2013), while during heat adaptation plasma volume expansion may increase $\dot{Q}$_max_. This view is supported by Kanstrup and Ekblom (1982), who demonstrated that an increase in PV by a 700 mL dextran infusion augments SV at rest and throughout exercise and therefore $\dot{Q}$. In terms of favorable changes in $\dot{V}$O_2max_ following heat training, there is still a lack of evidence in the literature (Travers et al., 2020; Baranauskas et al., 2021).

Concerning the regulation of SV and thus $\dot{Q}$_max_, our results support the hypothesis that total BV seems to be the major determinant of the SV response during exercise. BV expansion occurring during long-term training periods (Schmidt et al., 1988) may therefore represent an important part of cardio-circulatory adaptation to physical training. On the other hand, it is also important to consider that absolute BV may sometimes be of limited value to determine its impact on hemodynamics during exercise. Even more important is the hemodynamically active blood volume, i.e., the proportion of the total BV that affects pressure, flow, and cardiac function. This hemodynamically active BV would allow a high SV_max_ and thus $\dot{Q}$_max_ despite a relatively small absolute BV (Martino et al., 2002). In any case, however, we assume that a large resting BV provides a beneficial precondition for a high hemodynamically active BV.

### Blood Volume and Heart Volume

When untrained subjects were matched to trained endurance athletes in terms of BV via dextran infusion, an increase in SV during exercise in the untrained group was observed, but these values were still lower than those in the trained athletes (Hopper et al., 1988). This finding indicates that BV alone cannot explain the differences in SV, suggesting that a larger HV has to accompany a larger BV (Bonne et al., 2014) which is clearly demonstrated by the strong relationship between BV and HV in this study (Figure 2B). To date, very few studies have investigated the relationship between BV and cardiac size in healthy adults. It is widely accepted that increased end-diastolic dimensions of the right and left ventricles, increased LVMM, and increased volume of the left atrium are now well-established hallmarks of what has been defined as the athlete’s heart (Hellsten and Nyberg, 2015). However, these structural parameters have rarely been correlated with the prevailing BV, and there do not exist any data on the relationship between HV and BV. The magnitude of the HV is approximately 15% of the BV, and in our study, too, a change in the BV was associated with an approx. 15% change in the HV, which indicates a similar adjustment in both structures. In addition, we found LVMM to be significantly correlated with resting BV. Since the hemodynamic changes that occur during exercise constitute the primary stimulus for cardiac remodeling (Hellsten and Nyberg, 2015), it is reasonable to assume that high BV again plays a major role in these adaptations. As already mentioned above and regarding BV_max_ and $\dot{Q}$_max_, the correlation between BV and HV applies regardless of the anthropometric conditions and thus represents the likely coupled adaptation processes to long-term training stimuli (see Figures 2B,E).

### Blood Volume Shifts

This is one of the very few studies in which exercise-induced decreases in total BV were quantified during exercise. While the percentage changes in BV were calculated based on the changes in [Hb] and hematocrit in previous studies (Dill et al., 1974), our results showed an absolute mean reduction of 567 mL in this population. These findings are larger than those found by Wilkerson et al. (1977) who found a mean reduction of 363 mL at $\dot{V}$O_2max_ but comparable with the 532 mL reduction reported by Kawabata et al. (2004). The decrease in plasma volume, which becomes obvious by the accompanying hemoconcentration, is generally due to a greater filtration rate (caused by an increase in blood pressure), sweat loss (which is of minor importance during short lasting bouts of exercise as in this study), and especially lactate accumulation and the breakdown of creatine phosphate within the muscle cell (Craig et al., 2008). The latter of which cause an increased osmotic gradient, that, in turn, leads to an influx of water into the intracellular and interstitial space (Böning and Maassen, 1983; Maassen and Böning, 2008), thereby increasing the intracellular volume by as much as 15% (Sjogaard et al., 1985). While we can confirm earlier results regarding the relationship between the total PV at rest and the amount shifted until maximum exercise (Kawabata et al., 2004), it remains unclear whether endurance-trained athletes shift more water into the intracellular space during exercise than untrained subjects. After all, we found no significant correlation between the percentage changes in BV and $\dot{V}$O_2max_ and no correlation with the maximum lactate concentrations, which are thought to be the main cause of these volume shifts (Maassen and Böning, 1984, 2008). This suggests that training status does not affect the amount of water shifted to the intracellular space during exercise.

Although one must assume that any decrease in circulating BV affects the SV response during exercise, the mean SV in this population increased from rest to 60% P_max_ and was not different between 60 and 100% P_max_. The dissociation between the SV and BV response from resting to submaximal values (Figure 2) can be explained by an increased venous return that by far outmatches the BV shifts (Berlin and Bakker, 2014). However, in regard to a lacking further increase in SV from submaximal to maximal exercise, we can only speculate if these volume shifts might have a potential negative effect. It would be of great interest if volume matched fluid compensation in the extent of the individual volume shifts, i.e., via plasma or dextran infusion during exercise would lead to a greater increase in SV and thus $\dot{Q}$_max_, hence increasing $\dot{V}$O_2max_.

### Fluid Shifts and Oxygen Transport

Last, not all physiological mechanisms during high-intensity exercise inevitably lead to the optimization of oxygen transport. In this study, the SpO_2_ decreased by approximately 6%, which corresponds to data from Gaston et al. (2016) who found heterogeneous decreases in SpO_2_ in trained and untrained subjects. Our data also indicate a significant decrease in SpO_2_ with increasing $\dot{V}$O_2max_. This might be due to a larger $\dot{Q}$_max_ in the trained subjects (see Table 4), resulting in higher pulmonary blood flow and impaired O_2_ diffusion in the lungs (Goodrich et al., 2018). This view is supported by the significant correlation between SpO_2_ and $\dot{Q}$_max_. Without considering the effects of hemoconcentration and the augmentation of CaO_2_ in addition to the known reduction in arterial O_2_ saturation, the reduction in BV may decrease $\dot{Q}$_max_ and thus the maximum oxygen transport. Regarding the results from the cross-sectional part of this study, the reduction in BV by 567 mL might have reduced $\dot{Q}$ by 1.6 L⋅min^–1^ and thereby $\dot{V}$O_2max_ by 133 mL⋅min^–1^ during the performance test. These considerations, however, assume that organ perfusion remains constant despite the reductions in BV, which needs further investigation that would require the inclusion of parameters such as mean arterial pressure or vascular resistance.

All previous considerations have assumed that a change in hemoconcentration is accompanied by a change in arterial oxygen content. In this study, [Hb] increased by 1.3 g⋅dL^–1^ (8.5%) until maximum exercise, which, in theory would lead to an increase in CaO_2_ by 1.8 mL⋅dL^1^. However, SpO_2_ simultaneously decreased by 6.1% so that the arterial O_2_ content even tended to increase (see Table 3). When comparing the amount of O_2_ that is transported per minute either with or theoretically without the effects of the fluid shifts on $\dot{Q}$_max_ and the corresponding hemoconcentrations, the result is 5,376 mL⋅min^–1^ (with hemoconcentration, see Table 3) and 5,208 mL⋅min^–1^ (without hemoconcentration), respectively. These findings indicate that the fluid shifts completely compensate for the decrease in SpO_2_ and the reduced $\dot{Q}$_max_. Similar to acute altitude effects, the hemoconcentration due to transient plasma volume shifts could, therefore, be interpreted as a physiological adjustment to maintain oxygen transport capacity, without compromising performance.

## Limitations

Since we only conducted linear regression analysis, we cannot confirm cause and effect relationships; however, we can draw general conclusions. For the calculation of the possible effects of blood volume changes during exercise, we partly use data from the regression analyses obtained in the cross-sectional study which is based on a very heterogeneous group of test subjects. Therefore, the results must be considered with caution, even though they certainly show physiological trends. Direct manipulations of BV must be applied to compare the effects of volume shifts with fluid compensated conditions on hemodynamic mechanisms. To determine whether the increase in hemoconcentration and consequently maintained CaO_2_ really are fully compensatory for the reductions in $\dot{Q}$ discussed here, additional data such as blood flow to working muscle, mean arterial pressure and vascular resistance (since blood pressure was not measured) should also be collected to draw a more holistic conclusion. Whether the compensated CaO_2_ due to hemoconcentration influences a regulatory feedback mechanism on the ${\dot{Q}}_{\text{max}}$, as considered possible by Calbet (2000), cannot be explained by the results of the present study.

The determination of cardiac output with the Innocor system is relatively precise during exercise testing in our lab with a TE of 4.9%, which is comparable to previous investigations (Fontana et al., 2009). However, it only allows hemodynamic measurements at limited time intervals, making it somewhat difficult to draw a comprehensive conclusion on the precise time course of physiologic regulations. This is of special importance in interpreting the four types of SV responses according to Vella and Robergs (2005). Depending on the time of measurement at least three measurements during exercise are necessary to identify the individual responses. Other systems that allow continuous hemodynamic measurements should therefore be considered in future studies.

## Conclusion

We found a strong correlation between BV_max_ and $\dot{Q}$_max_, suggesting that an increase in BV_max_ by 1 L is associated with an increase in $\dot{Q}$_max_ by ∼3.5 L⋅min^–1^. Additionally, HV was closely correlated with BV_rest_, indicating that a change in BV by 1 L is correlated with a change in HV by 179 mL. Mean BV significantly decreased until maximum exercise by 567 mL. We hypothesize that the exercise-induced reductions in total BV may have detrimental effects on the $\dot{Q}$_max_ response, as this value might have changed by as much as 1.6 L⋅min^–1^ in our study. On the other hand, [Hb] increased by 1.3 g⋅dL^–1^ due to fluid shifts, thereby maintaining CaO_2_, which completely compensates for the exercise induced arterial desaturation. Our results support the general conviction that a high BV at rest is needed in order to achieve a high $\dot{Q}$_max_. Blood volume shifts during intense exercise may have a detrimental effect on $\dot{Q}$, but at the same time, they exert a beneficial effect on the oxygen transport system. This effect may maintain a reasonable avDO_2_, and thereby overcompensates for the negative effects on $\dot{Q}$_max_.

## Data Availability Statement

The raw data supporting the conclusions of this article will be made available by the authors, without undue reservation.

## Ethics Statement

The studies involving human participants were reviewed and approved by the Ethics Committee of the University of Bayreuth, Germany. The patients/participants provided their written informed consent to participate in this study.

## Author Contributions

All authors contributed to the data collection, analysis and interpretation of the data, drafting, and revising the manuscript, and approved the final version of the manuscript. The original study design was made by JS and WS and discussed with the other authors.

## Conflict of Interest

The results of this study are presented clearly, honestly and without fabrication, falsification, or inappropriate data manipulation. This study was financially supported by regular funds from the University of Bayreuth. WS was a managing partner of the Blood tec GmbH company, but he is unaware of any direct or indirect conflicts of interest with the contents of this manuscript. The remaining authors declare that the research was conducted in the absence of any commercial or financial relationships that could be construed as a potential conflict of interest.

## References

[B1] Baggish A. L. Wood M. J. (2011). Athlete’s heart and cardiovascular care of the athlete: scientific and clinical update. *Circulation* 123 2723–2735. 10.1161/CIRCULATIONAHA.110.981571 21670241

[B2] Baranauskas M. N. Constantini K. Paris H. L. Wiggins C. C. Schlader Z. J. Chapman R. F. (2021). Heat versus altitude training for endurance performance at sea level. *Exerc. Sport Sci. Rev.* 49 50–58. 10.1249/JES.0000000000000238 33044330

[B3] Bassett D. Howley E. (2000). Limiting factors for maximum oxygen uptake and determinants of endurance performance. *Med. Sci. Sports Exerc.* 32 70–84. 10.1097/00005768-200001000-00012 10647532

[B4] Berlin D. A. Bakker J. (2014). Understanding venous return. *Int. Care Med.* 40 1564–1566. 10.1007/s00134-014-3379-4 24966066

[B5] Böning D. Maassen N. (1983). Blood osmolality in vitro: dependence on PCO2, lactic acid concentration, and O2 saturation. *J. Appl. Physiol.* 54 118–122. 10.1152/jappl.1983.54.1.118 6402467

[B6] Bonne T. Doucende G. Flück D. Jacobs R. A. Nordsborg N. B. Robach P. (2014). Phlebotomy eliminates the maximal cardiac output response to six weeks of exercise training. *Am. J. Physiol.* 306 R752–R760. 10.1152/ajpregu.00028.2014 24622974

[B7] Calbet J. A. (2000). Oxygen tension and content in the regulation of limb blood flow. *Acta Physiol. Scand.* 168 465–472. 10.1046/j.1365-201x.2000.00698.x 10759583

[B8] Craig S. K. Byrnes W. C. Fleck S. J. (2008). Plasma volume during weight lifting. *Int. J. Sports Med.* 29 89–95. 10.1055/s-2007-965108 17960510

[B9] Daussin F. Ponsot E. Dufour S. Lonsdorfer-Wolf E. Doutreleau S. Geny B. (2007). Improvement of VO2max by cardiac output and oxygen extraction adaptation during intermittent versus continuous endurance training. *Eur. J. Appl. Physiol.* 101 377–383. 10.1007/s00421-007-0499-3 17661072

[B10] Dickhuth H. Urhausen A. Huonker M. Heitkamp H. Kindermannm W. Simon G. (1990). Echocardiographic determination of the heart size in sports medicine. *German J. Sportsmed.* 41 4–12.

[B11] Dill D. Braithwaite K. Adams W. Bernauer E. (1974). Blood volume of middle-distance runners: effect of 2,300-m altitude and comparison with non-athletes. *Med. Sci. Sports Exerc.* 6 1–7. 10.15373/22778179/may2014/193 4826686

[B12] Dill D. Costill D. (1974). Calculation of percentage changes in volumes of blood, plasma, and red cells in dehydration. *J. Appl. Physiol.* 37 247–248. 10.1152/jappl.1974.37.2.247 4850854

[B13] Eastwood A. Hopkins W. G. Bourdon P. C. Withers R. T. Gore C. J. (2008). Stability of hemoglobin mass over 100 days in active men. *J. Appl. Physiol.* 104 982–985. 10.1152/japplphysiol.00719.2007 18218904

[B14] Ekblom B. Hermansen L. (1968). Cardiac output in athletes. *J. Appl. Physiol.* 25 619–625. 10.1152/jappl.1968.25.5.619 4879852

[B15] Fontana P. Boutellier U. Toigo M. (2009). Reliability of measurements with innocor during exercise. *J. Sports Med.* 30 747–753. 10.1055/s-0029-1225340 19642059

[B16] Fricke G. (1965). About the behaviour of the cell factor during physical work. Determinations with T-1824 (Evans blue) and radioactive chromate. *Cardiologia* 47 25–44. 5867799

[B17] Gaston A.-F. Durand F. Roca E. Doucende G. Hapkova I. Subirats E. (2016). Exercise-induced hypoxaemia developed at sea-level influences responses to exercise at moderate altitude. *PLoS One* 11:e0161819. 10.1371/journal.pone.0161819 27583364 PMC5008680

[B18] González-Alonso J. Mora-Rodríguez R. Coyle E. F. (2000). Stroke volume during exercise: interaction of environment and hydration. *Am. J. Physiol. Heart Circul. Physiol.* 278 H321–H330. 10.1152/ajpheart.2000.278.2.H321 10666060

[B19] Goodrich J. A. Ryan B. J. Byrnes W. C. (2018). The Influence of oxygen saturation on the relationship between hemoglobin mass and VO 2 max. *Sports Med. Int. Open* 2 E98–E104. 10.1055/a-0655-7207 30539125 PMC6225968

[B20] Gore C. J. Bourdon P. C. Woolford S. M. Ostler L. M. Eastwood A. Scroop G. C. (2006). Time and sample site dependency of the optimized co-rebreathing method. *Med. Sci. Sports Exerc.* 38 1187–1193. 10.1249/01.mss.0000222848.35004.41 16775562

[B21] Heinicke K. Wolfarth B. Winchenbach P. Biermann B. Schmid A. Huber G. (2001). Blood volume and hemoglobin mass in elite athletes of different disciplines. *Physiol. Biochem.* 22 504–512. 10.1055/s-2001-17613 11590477

[B22] Hellsten Y. Nyberg M. (2015). Cardiovascular adaptations to exercise training. *Comprehensive Physiol.* 6 1–32. 10.1002/cphy.c140080 26756625

[B23] Higginbotham M. Morris K. Williams R. (1986). Regulation of stroke volume during submaximal and maximal upright exercise in normal man. *Circ. Res.* 58 281–291. 10.1161/01.res.58.2.281 3948345

[B24] Hopper M. Coggan A. Coyle E. (1988). Exercise stroke volume relative to plasma-volume expansion. *J. Appl. Physiol.* 64 404–408. 10.1152/jappl.1988.64.1.404 2451658

[B25] Hütler M. Beneke R. Böning D. (2000). Determination of circulating hemoglobin mass and related quantities by using capillary blood. *Med. Sci. Sports Exerc.* 32 1024–1027. 10.1097/00005768-200005000-00022 10795796

[B26] Kanstrup I. Ekblom B. (1982). Acute hypervolemia, cardiac performance, and aerobic power during exercise. *J. Appl. Physiol.* 52 1186–1191. 10.1152/jappl.1982.52.5.1186 7096143

[B27] Kawabata T. Suzuki T. Miyagawa T. (2004). Effect of blood volume on plasma volume shift during exercise. *J. Ther. Biol.* 29 775–778. 10.1016/j.jtherbio.2004.08.054

[B28] La Sanz-de Garza M. Carro A. Caselli S. (2020). How to interpret right ventricular remodeling in athletes. *Clin. Cardiol.* 43 843–851. 10.1002/clc.23350 32128858 PMC7403694

[B29] Levine B. D. (2008). VO2max: what do we know, and what do we still need to know? *J. Physiol.* 586 25–34. 10.1113/jphysiol.2007.147629 18006574 PMC2375567

[B30] Lundby C. Montero D. Joyner M. (2017). Biology of VO2 max: looking under the physiology lamp. *Acta Physiol.* 220 218–228. 10.1111/apha.12827 27888580

[B31] Maassen N. Böning D. (1984). “Arbeitsbedingte hämokonzentration und osmolalität,” in *Stellenwert der Sportmedizin in Medizin und Sportwissenschaft/Position of Sports Medicine in Medicine and Sports Science*, ed. Jeschke D. (Berlin: Springer-Verlag), 93–99.

[B32] Maassen N. Böning D. (2008). Physiological side-effects of lactic acid. *German J. Sportsmed.* 59 292–296.

[B33] Magder S. (2016). Volume and its relationship to cardiac output and venous return. *Crit. Care* 20:271. 10.1186/s13054-016-1438-7 27613307 PMC5018186

[B34] Martino M. Gledhill N. Jamnik V. (2002). High VO2max with no history of training is primarily due to high blood volume. *Med. Sci. Sports Exerc.* 34 966–971. 10.1097/00005768-200206000-00010 12048323

[B35] Munch G. D. W. Svendsen J. H. Damsgaard R. Secher N. H. González-Alonso J. Mortensen S. P. (2014). Maximal heart rate does not limit cardiovascular capacity in healthy humans: insight from right atrial pacing during maximal exercise. *J. Physiol.* 592 377–390. 10.1113/jphysiol.2013.262246 24190933 PMC3922500

[B36] Patel A. J. Wesley R. Leitman S. F. Bryant B. J. (2013). Capillary versus venous haemoglobin determination in the assessment of healthy blood donors. *Vox Sanguinis* 104 317–323. 10.1111/vox.12006 23294266 PMC3633672

[B37] Prommer N. Schmidt W. (2007). Loss of CO from the intravascular bed and its impact on the optimised CO-rebreathing method. *Eur. J. Appl. Physiol.* 100 383–391. 10.1007/s00421-007-0439-2 17394011

[B38] Roach R. C. Koskolou M. D. Calbet J. A. Saltin B. (1999). Arterial O2 content and tension in regulation of cardiac output and leg blood flow during exercise in humans. *Am. J. Physiol.* 276 H438–H445. 10.1152/ajpheart.1999.276.2.H438 9950843

[B39] Robertson E. Saunders P. Pyne D. Gore C. Anson J. (2010). Effectiveness of intermittent training in hypoxia combined with live high/train low. *Eur. J. Appl. Physiol.* 110 379–387. 10.1007/s00421-010-1516-5 20503055

[B40] Rowell L. B. (1986). “Circulatory adjustments to dynamic exercise,” in *Human Circulation Regulation during Physical Stress*, ed. Rowell L. B. (New York, NY: Oxford University Press), 213–256.

[B41] Rowland T. (2005). Circulatory responses to exercise: are we misreading Fick? *Chest* 127 1023–1030. 10.1378/chest.127.3.1023 15764789

[B42] Rowland T. (2009). Endurance athletes’ stroke volume response to progressive exercise. a critical review. *Sports Med.* 39 687–695. 10.2165/00007256-200939080-00005 19769416

[B43] Schmidt W. Brabant G. Kröger C. Strauch S. Hilgendorf A. (1990). Atrial natriuretic peptide during and after maximal and submaximal exercise under normoxic and hypoxic conditions. *Eur. J. Appl. Physiol.* 61 398–407. 10.1007/bf00236059 2150372

[B44] Schmidt W. Maassen N. Trost F. Böning D. (1988). Training induced effects on blood volume, erythrocyte turnover and haemoglobin oxygen binding properties. *Eur. J. Appl. Physiol.* 57 490–498. 10.1007/bf00417998 3396563

[B45] Schmidt W. Prommer N. (2005). The optimised CO-rebreathing method: a new tool to determine total haemoglobin mass routinely. *Eur. J. Appl. Physiol.* 95 486–495. 10.1007/s00421-005-0050-3 16222540

[B46] Siebenmann C. Hug M. Keiser S. Müller A. van Lieshout J. Rasmussen P. (2013). Hypovolemia explains the reduced stroke volume at altitude. *Physiol. Rep.* 1:e00094. 10.1002/phy2.94 24303166 PMC3841030

[B47] Sjogaard G. Adams R. Saltin B. (1985). Water and ion shifts in skeletal muscle of humans with intense dynamic knee extension. *Am. J. Physiol.* 248(Pt. 2) R190–R196. 3970234 10.1152/ajpregu.1985.248.2.R190

[B48] Skattebo Ø Bjerring A. Auensen M. Sarvari S. I. Cumming K. T. Capelli C. (2020). Blood volume expansion does not explain the increase in peak oxygen uptake induced by 10 weeks of endurance training. *Eur. J. Appl. Physiol.* 120 985–999. 10.1007/s00421-020-04336-2 32172291 PMC7181565

[B49] Stöhr E. J. González-Alonso J. Shave R. (2011). Left ventricular mechanical limitations to stroke volume in healthy humans during incremental exercise. *Am. J. Physiol. Heart Circ. Physiol.* 301 H478–H487. 10.1152/ajpheart.00314.2011 21572016

[B50] Travers G. González-Alonso J. Riding N. Nichols D. Shaw A. Périard J. D. (2020). Exercise heat acclimation has minimal effects on left ventricular volumes, function and systemic hemodynamics in euhydrated and dehydrated trained humans. *Am. J. Physiol. Heart Circ. Physiol.* 319 H965–H979. 10.1152/ajpheart.00466.2020 32886001

[B51] Vella C. A. Robergs R. A. (2005). A review of the stroke volume response to upright exercise in healthy subjects. *Br. J. Sports Med.* 39 190–195. 10.1136/bjsm.2004.013037 15793084 PMC1725174

[B52] Warburton D. Haykowsky M. Quinney H. Blackmore D. Teo K. Humen D. (2002). Myocardial response to incremental exercise in endurance-trained athletes: influence of heart rate, contractility and the Frank-Starling effect. *Exp. Physiol.* 87 613–622. 10.1113/eph8702372 12481936

[B53] Wilkerson J. E. Gutin B. Horvath S. M. (1977). Exercise-induced changes in blood, red cell, and plasma volumes in man. *Med. Sci. Sports Exerc.* 9 155–158. 593077

[B54] Wilmore J. H. Stanforth P. R. Gagnon J. Rice T. Mandel S. Leon A. S. (2001). Cardiac output and stroke volume changes with endurance training: the HERITAGE family study. *Med. Sci. Sports Exerc.* 33 99–106. 10.1097/00005768-200101000-00016 11194119

[B55] Woodiwiss A. Norton G. (1995). Exercise-induced cardiac hypertrophy is associated with an increased myocardial compliance. *J. Appl. Physiol.* 78:1311. 10.1152/jappl.1995.78.4.1303 7615437

